# Benefits of exercise on cognitive impairment in alcohol use disorder following alcohol withdrawal

**DOI:** 10.1002/2211-5463.13865

**Published:** 2024-07-25

**Authors:** Zhen Lyu, Zhi‐Gang Gong, Min‐Xia Huang, Si‐Ping Xin, Mao‐Zhong Zou, Yu‐Quan Ding

**Affiliations:** ^1^ Key Lab of Aquatic Sports Training Monitoring and Intervention of General Administration of Sport of China, Faculty of Physical Education Jiangxi Normal University Nanchang China; ^2^ School of Psychology Shanghai University of Sport China; ^3^ Science and Technology College of Nanchang Hangkong University Jiujiang China

**Keywords:** alcohol use disorder, alcohol vapor exposure, exercise, learning and memory, monoamine neurotransmitters

## Abstract

Although most cognitive impairments induced by prolonged alcohol consumption tend to improve within the initial months of abstinence, there is evidence suggesting certain cognitive deficits may persist. This study aimed to investigate the impact of aerobic exercise on learning and memory in alcohol use disorder (AUD) mice following a period of abstinence from alcohol. We also sought to assess the levels of monoamine neurotransmitters in the hippocampus. To this end, we established an AUD mouse model through a two‐bottle choice (sucrose fading mode and normal mode) and chronic intermittent alcohol vapor (combined with intraperitoneal injection) and randomly allocated mice into exercise groups to undergo treadmill training. Learning and memory abilities were assessed through the Morris water maze test and spontaneous activity was evaluated using the open field test. The levels of dopamine, norepinephrine, serotonin, and brain‐derived neurotrophic factor in the hippocampus were quantified using enzyme‐linked immunoassay (ELISA) kits. The findings reveal that after cessation of alcohol consumption, learning and memory abilities in AUD mice did not completely return to normal levels. The observed enhancement of cognitive functions in AUD mice through aerobic exercise may be attributed to restoring levels of monoamine neurotransmitters in the hippocampus, boosting brain‐derived neurotrophic factor (BDNF) concentrations, and facilitating an increase in hippocampal mass. These results offer empirical evidence to support aerobic exercise as a viable therapeutic strategy to alleviate cognitive deficits associated with AUD.

## Graphical abstract

The article investigates the impact of aerobic exercise on learning and memory in mice with alcohol use disorder following a period of abstinence from alcohol. It elucidates the relationship between cognitive abilities and molecular mechanisms, with a specific focus on assessing the levels of monoamine neurotransmitters in the hippocampus.

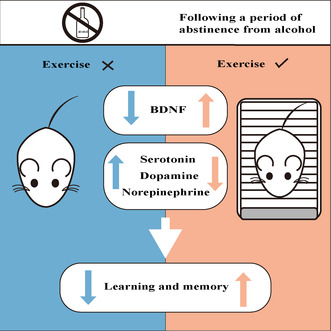

**Abbreviations**
5‐HT: serotonin
AUD: alcohol use disorder
BAC: blood alcohol concentration
BDNF: brain‐derived neurotrophic factor
CIAV: chronic intermittent alcohol vapor
CORT: corticosterone
DA: dopamine
ELISA: enzyme‐linked immunosorbent assay
HPA axis: hypothalamus‐pituitary–adrenal axis
HRP: horseradish peroxidase
LC: locus coeruleus
MWM: Morris water maze
NE: norepinephrine
OFT: open field test
TP: total protein content
VTA: ventral tegmental area
β‐EP: β‐endorphin

Alcohol use disorder (AUD) is a neuropsychiatric disorder encompassing behavioral, neurobiological, and psychosocial components [1]. It can be classified into alcohol dependence and alcohol abuse [2]. Although cognitive impairment resulting from chronic alcohol consumption in individuals with alcohol abuse tends to improve within a few months of abstinence [3], research has indicated that certain cognitive deficits may persist even after long‐term abstinence [4]. Moreover, excessive alcohol consumption during both short‐term and long‐term periods of abstinence is linked to multiple cognitive deficits. Impairments in attention, working memory, processing speed, visuospatial abilities, executive functions, impulsivity, learning, memory, and language fluency have all been shown to be affected in alcohol addiction [5]. Therefore, mere abstinence is insufficient for the restoration of cognition, necessitating additional interventions to facilitate a return to normal levels.

Exercise, as a noninvasive therapeutic approach, has demonstrated widespread efficacy in enhancing cognitive functions [6] and has been proven to be acceptable for patients undergoing treatment for alcohol dependence [7]. Furthermore, exercise has been shown to possess both therapeutic and protective effects against alcohol‐induced brain damage [8]. Extensive research has consistently demonstrated significant enhancements in hippocampal function following long‐term regular physical activity [9, 10]. Moreover, regular exercise can facilitate neuroplasticity processes such as synaptic labeling and neurogenesis in models of chronic alcohol‐induced hippocampal deterioration [11, 12]. Therefore, exercise can serve as an intervention to facilitate hippocampal repair following alcohol‐induced damage.

In recent years, there has been considerable attention focused on the impact of monoamine neurotransmitters on learning and memory. These chemical messengers play a crucial role in transmitting information between neurons and exert significant influence on cognitive behavior [13]. It is well known that the hippocampus has important connections to cognitive behavior. Some evidence suggested that monoamine neurotransmitters including dopamine (DA), norepinephrine (NE), and serotonin (5‐HT) can affect hippocampal function, which results in changes in learning and memory abilities [14, 15, 16]. Additionally, they have been associated with substance dependence and depression [17]. At present, the most extensively investigated monoamine neurotransmitters in the field of substance dependence are DA, NE, and 5‐HT. To be specific, animal experiments revealed that nicotine induces alterations in ventral tegmental area (VTA) dopaminergic neurons, leading to inhibitory synaptic transmission and excessive excitability [18]. It has been reported that exposure to the drug leads to activation of the hypothalamus–pituitary–adrenal (HPA) axis mediated by corticosterone (CORT), which subsequently accelerates NE synthesis and elevates intrahippocampal NE/5‐HT levels. This pathway is thought to be an important neural circuit for inducing drug craving and relapse behavior [19]. Furthermore, disrupted homeostasis of both 5‐HT and DA has been observed in the central nervous system of AUD [20]. These investigations collectively demonstrate the crucial association between monoamine neurotransmitters and alcohol use disorders. However, there is a paucity of studies examining the impact of monoamine neurotransmitters on learning and memory in the context of AUD.

Therefore, the objective of this study was to investigate the impact of aerobic exercise on learning and memory in mice with AUD following alcohol withdrawal. Furthermore, it aimed to elucidate the underlying mechanism by assessing hippocampal levels of monoamine neurotransmitters.

## Materials and methods

### Animals

Fifty‐two male C57BL/6J mice, aged 7 weeks, were procured from Hunan SJA Laboratory Animal Co., Ltd. (Changsha, Hunan, China). The mice were individually housed for 9 weeks (1 week of acclimation and 8 weeks of two‐bottle choices) and subsequently group‐housed for the remaining duration of the experiment. Upon arrival, the mice had access to food and water *ad libitum* and were maintained on a 12‐h dark‐light cycle (from 14:00 to 02:00 h). All experimental procedures were conducted in accordance with the NIH guidelines and approved by the Experimental Animal Ethics and Welfare Committee of the College of Life Sciences, Jiangxi Normal University (No. 20210318‐003).

After 1 week of acclimation, 12 mice were randomly selected as sentinel animals. The remaining participants were then allocated into two groups: alcohol use disorder (AUD) and control (Con) groups (*n* = 20). This allocation was based on body weight, following a randomized block experimental design [21]. At the completion of AUD induction (alcohol drinking and chronic intermittent alcohol exposure), the mice were further divided into four groups: alcohol use disorder and sedentary (AUD‐Sed) group, alcohol use disorder and exercise (AUD‐Ex) group, control sedentary (Con‐Sed) group, and control exercise (Con‐Ex) group, following the above design (*n* = 10). Notably, there was no statistically significant difference in alcohol consumption between the two AUD groups.

### AUD model

#### Alcohol consumption

**Fig. 1:**
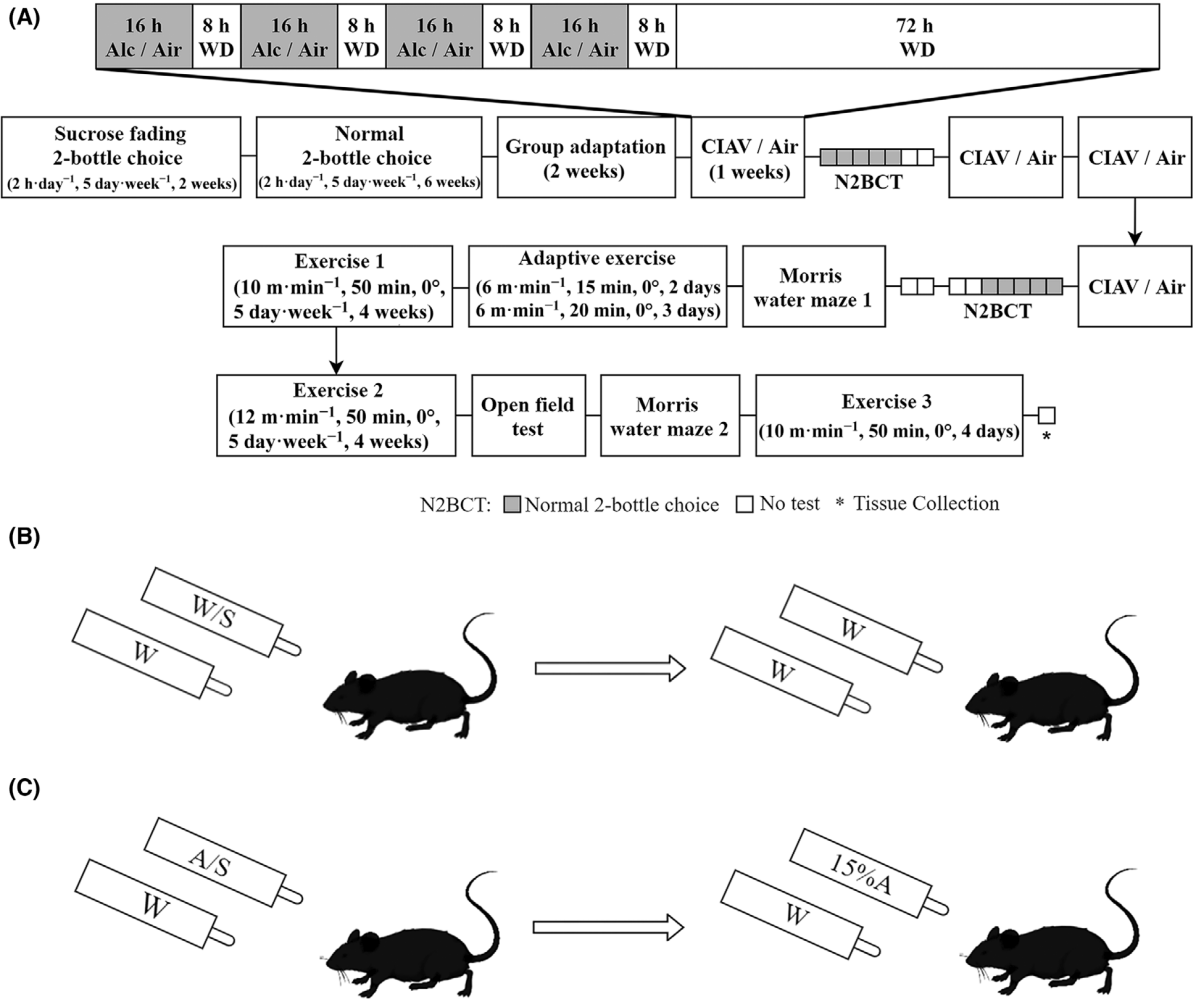
Experimental flowchart and schematic of two‐bottle choice. (A) The experimental flowchart. The CIAV, N2BCT, WD, respectively, represent chronic intermittent alcohol vapor exposure, normal two‐bottle choice test, and alcohol withdrawal process. (B) The Con group transforms from sucrose fading two‐bottle choice to normal two‐bottle choice. (C) The AUD group transforms from sucrose fading two‐bottle choice to normal two‐bottle choice. The 2‐h drinking sessions started at 13:30 h from Monday to Friday for a duration of 8 weeks, including sucrose fading two‐bottle choices and normal two‐bottle choices. In the sucrose fading two‐bottle choice procedure for the AUD group, the bottle alcohol/sucrose (A/S) was prepared with the following concentrations: 10%A/5%S, 12%A/5%S, 15%A/5%S, 15%A/2%S, 15%A/1%S. The mice in the AUD group were given each concentration for 2 days, 5 days a week, for 2 weeks. In the normal two‐bottle alcohol drinking choice procedure, the bottle 15%A contains a fixed 15% alcohol solution without sucrose. The mice in the AUD group drank for 5 days per week for 6 weeks. The procedure for the Con group remained the same as that for the AUD group, except that the bottles were not mixed with alcohol. The 95% alcohol (Xintai Huayuan Medical and Health Products Co., Ltd., Xintai City, Shandong Province, China) and sucrose (Sangon Biotech Co., Ltd., Shanghai city, China) were mixed daily with distilled water to achieve the appropriate alcohol (v/v) and sucrose (w/v) concentrations, respectively. The bottle W contained the same volume of distilled water.

The mice were trained to consume alcohol in their home cages under a limited access, two‐bottle choice paradigm, as previously described [22]. Briefly, a modified sucrose fading procedure was employed with a final solution containing 15% (v/v) alcohol. For further details, please refer to Fig. 1B,C. The mice consumed water and alcohol separately from 15 mL bottles. Two‐hour drinking sessions commenced at 13:30 h from Monday to Friday for a duration of 8 weeks. These sessions encompassed sucrose fading two‐bottle choices and standard two‐bottle choices, with daily recording of alcohol consumption and weekly monitoring of mice weights. To prevent any side preference, the positions of alcohol and water bottles were randomly interchanged throughout the study. The measurement of water consumption was omitted due to minimal intake, as previously reported [23]. Notably, inaccuracies were observed in the readings of alcohol consumption during weeks 5–8 due to residual alcohol in the 15 mL drinking bottle. Consequently, data from this period were excluded from the analysis. To ensure accurate measurement of alcohol consumption, bottles were weighed at Week 1 and Week 4 following vapor exposure.

Following 8 weeks of two‐bottle choices, the mice were changed from individually housing to group‐housing.

#### Chronic intermittent alcohol vapor exposure

Following 2 weeks of group adaptation, the chronic intermittent alcohol vapor (CIAV) exposure phase involved a combination of intraperitoneal alcohol injections and alcohol vapor inhalation, conducted according to pre‐established protocols [23, 24, 25]. Briefly, the AUD and sentinel mice (only monitoring blood alcohol concentration in CIAV) were exposed to alcohol vapor in Plexiglas inhalation chambers for 16 h (from 18:00 to 10:00), followed by 8 h in their home cages. This process was repeated for 4 consecutive days, followed by 3 days in their home cages. The mice were returned to their home cages in a standard environment with ambient air, and all items such as food, water bottles, and bedding were replaced during the withdrawal period. The control group mice underwent similar handling procedures but were exposed to air in control chambers (Fig. 1A).

Prior to each 16‐h exposure, both AUD and sentinel mice received a 1.6 g·kg^−1^ alcohol solution to achieve and maintain a stable blood alcohol concentration (BAC) level. The corresponding control mice were administered an equivalent volume of 0.9% saline solution. In this study, to prevent any potential cognitive impairments, the mice did not receive pyrazole, an alcohol dehydrogenase inhibitor known to exert side effects by inhibiting the central nervous system [26] and affecting mitochondrial function [27]. Both the pre‐experiment and Shang *et al*. [27] observed minimal variation in BAC following intraperitoneal (i.p.) administration at the same alcohol vapor concentration. The alcohol vapor exposure system effectively maintains BAC levels within the range of 175–225 mg·dL^−1^, as determined by the pre‐experiment. Submandibular vein blood was immediately collected from six sentinel mice at the conclusion of weekly alcohol vapor exposure (250 μL; the same mice were not collected at adjacent blood collection times) for measurement of BAC using the QuantiChrom^TM^ Ethanol Assay Kit (BioAssay Systems Inc., Northern California, USA). The alcohol vapor detector (Shenzhen Shenguoan Electronic Co., Ltd., Shenzhen city, Guangdong Province, China) was utilized multiple times per day during the alcohol exposure period to monitor the targeted concentration of alcohol vapor. CIAV is a widely recognized classical AUD modeling protocol [24, 28]. It has been shown to induce increased alcohol consumption in mice [25]. To mitigate the potential impact of age‐related cognitive decline, alcohol consumption in AUD mice was evaluated exclusively at the culmination of both Week 1 and Week 4 of vapor exposure using a standardized two‐bottle choice paradigm.

### Exercise intervention

The running speed (75% VO_2 max_) on the treadmill (Anhui Zhenghua Biological Apparatus Facilities Co., Ltd., Huaibei City, Anhui Province, China) was determined based on a study conducted by Fernando *et al*. [29]. The protocol was adjusted following the methods described by He *et al*. [30] and Yu *et al*. [31]. Further details can be found in Table 1. The mice in the Ex‐groups underwent a 9‐week aerobic exercise intervention, comprised of an initial adaptive exercise week followed by 8 weeks of formal exercise. Each intervention session commenced at 16:00 h. Additionally, in order to mitigate potential attenuation of the exercise effect on mice, exercise was reintroduced subsequent to the completion of the Morris water maze test due to its prolonged duration. These mice were subjected to 4 days aerobic exercise (10 m·min^−1^, 50 min, 0 incline). In contrast, mice in the Sed‐groups were placed on the treadmill without engaging in any physical activity. During the period of exercise intervention, the animals were not housed in isolation.

**Table 1:** Exercise intervention program. The Ex‐groups exercised 5 days a week, with two rest days. They underwent a 5‐min warm‐up and relaxation exercises at a speed of 4 m·min^−1^ before and after each training session, respectively.

Program	Adaptive exercise (Week 1)		Formal exercise (Week 2–9)	
Week	Week 1		Week 2–5	Week 6–9
Time (days)	1–2	3–5	1–5	1–5
Speed (m·min^−1^)	6	6	10	12
Running time (min)	15	20	50	50
Incline	0	0	0	0

### Behavioral testing

#### Open field test

Following the methodology described by Krasnova *et al*. [32], mice were gently transferred from their home cages to the central area of the experimental chamber for conducting the open field test (OFT). The experimenter promptly exited, allowing the mice to freely explore for a duration of 5 min. Subsequently, the mice were returned to their respective home cages.

Automated animal behavior evaluation software was utilized to record and analyze the movements of mice. The experimental chambers were disinfected with 75% alcohol by the same experimenter to eliminate any potential odor, and left to air dry. Mice were received on the day following completion of formal exercise.

#### Morris water maze test

The Morris water maze (MWM) test was adapted from the study by Bromley‐Brits *et al*. [33] with appropriate modifications, in accordance with established protocols. MWM entails a circular pool measuring 1.2 m in diameter and 0.52 m in depth, equipped with a camera system to track animal movement trajectories. The circular pool was enclosed by opaque curtains, while the interior of the pool was uniformly black, divided into four quadrants of equal size. A removable escape platform, measuring 9 cm in diameter, was positioned 1 cm below the water surface within the third quadrant.

Due to the black fur of the mice, an odorless and nontoxic food whitening agent (titanium dioxide; Shanghai Jiang Hu Titanium White Product Co., Ltd., Shanghai city, China) was added to the water in order to facilitate accurate recording and analysis of the swimming track. Additionally, a white cloth was used to cover the escape platform. The spatial cues consisted of eight shapes, including four rectangular figures in different colors (blue, green, red, pink) and four white figures in different shapes (circle, triangle, square, pentagram). These color‐shape combinations were consistently displayed together at the center of each quadrant on the wall (e.g. both a blue rectangular figure and a white square figure were positioned at the first quadrant).

The MWM protocol comprised a 5‐day training phase followed by a 1‐day spatial probe test. During the training phase, mice were subjected to testing with a hidden platform and recorded both the escape latency and swimming velocity. Each day, they underwent four training sessions in which they were placed in the water from four different quadrants, sequentially facing the wall of the pool. The duration of each training session was 60 s. The escape latency was defined as the time required for the mice to reach the platform, and if the mice autonomously located the platform within 60 s, they were allowed to remain on it for an additional 15 s and subsequently recorded the escape latency. If they failed to find it within 60 s, they were guided to it and allowed to stay there for 15 s. The measured escape latency was recorded as 60 s. On Day 6, the mice underwent a spatial probe test without a platform, following the four training sessions described above. This test recorded both the number of target platform location crossings and swimming velocity. The MWM test 1 was conducted 4 days after the last session of alcohol consumption, while the MWM test 2 took place on the day following completion of OFT.

### Hippocampal tissues collection

The hippocampal tissues were collected 24 h after the final exercise session to mitigate potential stress‐induced effects from exercise. Groups of mice were dissected alternately to ensure simultaneous sample collection across all groups. The sampling procedure is outlined as follows: (a) The mice were anesthetized using inhalation of CO_2_ and euthanized by the spinal cord dislocation method, followed by whole‐brain removal. (b) The hippocampus was carefully dissected from the cerebral cortex and surrounding brain tissue by the same experimenter under a chilled condition. The tissues were thoroughly rinsed with 4 °C, 0.9% saline solution to ensure cleanliness, while ensuring that the tissue surface remained dry to prevent any contamination during subsequent testing procedures. (c) The hippocampal tissues were weighed and promptly transferred into cryotubes for immediate flash‐freezing using liquid nitrogen, followed by storage at −80 °C as a contingency for subsequent assays.

### ELISA

The total protein content (TP) in the hippocampal tissue was measured using the Bicinchoninic Acid method (BCA method). Dopamine (DA), serotonin (5‐HT), norepinephrine (NE), and brain‐derived neurotrophic factor (BDNF) were all quantified using enzyme‐linked immunosorbent assay (ELISA) methods. The units for TP are μg·mL^−1^, for DA and BDNF are pg·mL^−1^, and for 5‐HT and NE are ng·mL^−1^. The assays were conducted strictly according to the instructions provided with the ELISA kits. The specific operational steps are as follows.

#### Tissue homogenization

The frozen hippocampal tissue was taken out from the −80 °C freezer and weighed accurately. It was then mixed with precooled phosphate‐buffered saline (PBS) buffer (pH 7.4) in a ratio of 1:9 by weight (g) to volume (mL), along with grinding steel balls. The mixture was then homogenized using a precooled low‐temperature homogenizer set at 4 °C, grinding for 30 s, pausing for 15 s, and repeated three times. After homogenization, the mixture was centrifuged in a precooled centrifuge at 4 °C, 14167 ***g***, for 10 min. The supernatant of the 10% tissue homogenate was used for ELISA kit analysis, and 5 μL of this supernatant was further diluted with precooled PBS buffer in a 1:9 ratio to create a 1% tissue homogenate supernatant for TP measurement. The remaining supernatant was kept at 4 °C for ELISA analysis.

#### TP measurement

(a) The working solution from the kit was prepared according to the manual and stored at 4 °C. Blank, standard, and test wells were set up in a 96‐well plate. (b) Distilled water, 524 μg·mL^−1^ standard solution, and 1% supernatant were added to the blank, standard, and test wells, respectively, with each well receiving 10 μL. (c) 250 μL of working solution was added to each well, and the plate was gently shaken to mix. (d) The plate was incubated at 37 °C for 30 min, then the absorbance at 562 nm was read using an ELISA reader. (e) The protein concentration of the samples (μg·mL^−1^) was calculated using the formula: (Test well absorbance − Blank well absorbance)/(Standard well absorbance − Blank well absorbance) × 524 μg·mL^−1^ × 100.

#### DA, 5‐HT, NE, and BDNF measurement

The operational steps for the DA, 5‐HT, NE, and BDNF ELISA kits were consistent and detailed as follows: (a) Preparation: The kits were taken out from the 4 °C refrigerator and allowed to reach room temperature before use. Washing buffer was diluted with distilled water at a ratio of 1:20. (b) After the kits reached room temperature, the necessary strips were taken out of the foil bag, with the remaining strips sealed in a self‐sealing bag and refrigerated at 4 °C. (c) Standard, blank, and sample wells were set up and recorded in the strips. Different concentrations of standards were added to the standard wells, and 10% supernatant was added to the sample wells, with each well receiving 50 μL. The blank wells received no addition. (d) Horseradish peroxidase (HRP)‐conjugated detection antibodies were added to the standard and sample wells, with each well receiving 100 μL. Blank wells received no antibodies. The wells were then sealed with a cover film and incubated at 37 °C for 60 min. (e) After incubation, the liquid was discarded, and the wells were patted dry on absorbent paper. Each well was filled with 350 μL washing buffer, allowed to stand for 1 min, then discarded and patted dry. This washing step was repeated five times. (f) 50 μL of color developing agents A and B were added to each well, gently shaken to mix, then incubated in a 37 °C dark environment for 15 min. (g) After incubation, 50 μL of stop solution was added to each well to terminate the reaction. (h) The absorbance at 450 nm (OD value) for each well was measured, a standard curve was plotted, and a linear regression equation obtained. The concentrations of the samples were calculated by substituting the OD values of the samples into the equation.

The concentrations of total protein were determined using the total protein assay kit (BCA method) provided by Nanjing Jiancheng Bioengineering Co., Ltd., Nanjing city, Jiangsu Province, China. Moreover, ELISA assay kits from Jiangsu Meimian Industrial Co., Ltd., Yancheng City, Jiangsu Province, China were employed for the measurement of DA, NE, 5‐HT, and BDNF. All experimental procedures strictly adhered to the instructions provided with each assay kit.

### Statistical analysis

The data were presented as mean ± SEM. For comparisons of alcohol consumption and the data from the spatial probe test in the MWM, *P*‐values were calculated using a two‐tailed Student's *t*‐test, as described in the Results section. The data from the training phase in MWM were analyzed using a repeated‐measures two‐way analysis of variance (ANOVA). The Mauchly's test of sphericity was employed, and the Greenhouse‐Geisser correction was applied when necessary. Subsequently, a two‐way ANOVA was conducted to analyze the remaining data in this study. Fischer's Least Significant Difference (LSD) *post‐hoc* comparisons were performed based on the results of the ANOVA analysis to identify significant differences, if applicable. Statistical significance was determined at *P* < 0.05. The data were analyzed using spss 26.0 statistical software (Chicago, IL, USA), and figures were generated using graphpad prism 9 (La Jolla, CA, USA).

## Results

### Increased alcohol consumption and maintained blood alcohol concentration by repeated CIAV

**Fig. 2:**
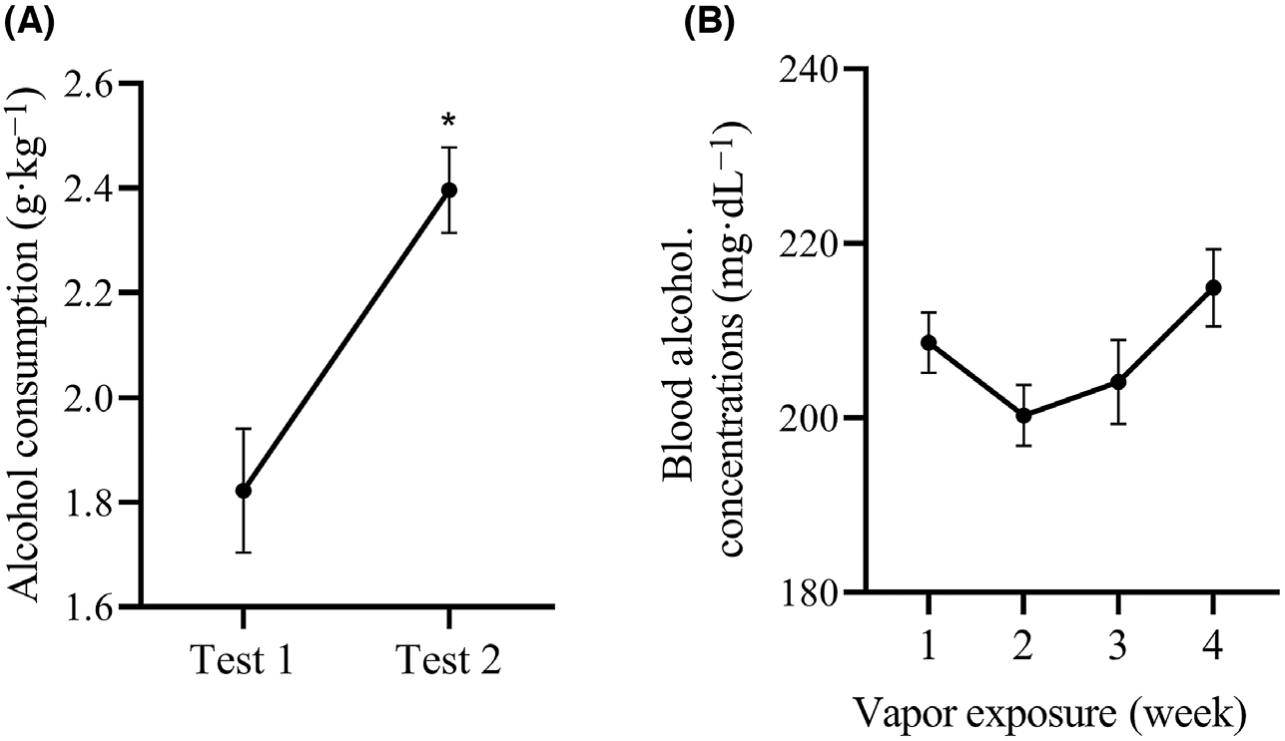
Increased alcohol consumption maintained stable blood alcohol concentrations during the period of chronic intermittent vapor exposure. (A) Changes in alcohol consumption were assessed in mice with alcohol use disorder (AUD). Test 1 represents the normal two‐bottle alcohol drinking choice test conducted at the end of week 1 of vapor exposure, while test 2 represents the same test conducted at the end of week 4 of vapor exposure. Alcohol consumption (g·kg^−1^) was calculated as alcohol intake (g) divided by mice weight (kg) (*n* = 20 per test). (B) Changes in blood alcohol concentrations were assessed in the sentinel mice. The mean BAC was maintained within the range of 200–220 mg·dL^−1^ (*n* = 6 per time; the blood was collected at nonadjacent times for the same mice). The data are represented as mean ± SEM and analyzed using a paired Student's *t*‐test. *P < 0.05 indicates statistical significance.

The mice in the AUD group were tested using the normal two‐bottle alcohol drinking choice at the end of week 1 and week 4 of CIAV, respectively. Upon comparing the results of test 1 and test 2, significant differences were observed in alcohol consumption (paired Student's *t*‐test, *t* = −4.589, *P* = 0.01), indicating that repeated CIAV led to an increased alcohol consumption (Fig. 2A). The mean BAC of sentinel mice maintained within the range of 200–220 mg·dL^−1^ (Fig. 2B).

### Diminished learning and memory in CIAV‐treated

**Fig. 3:**
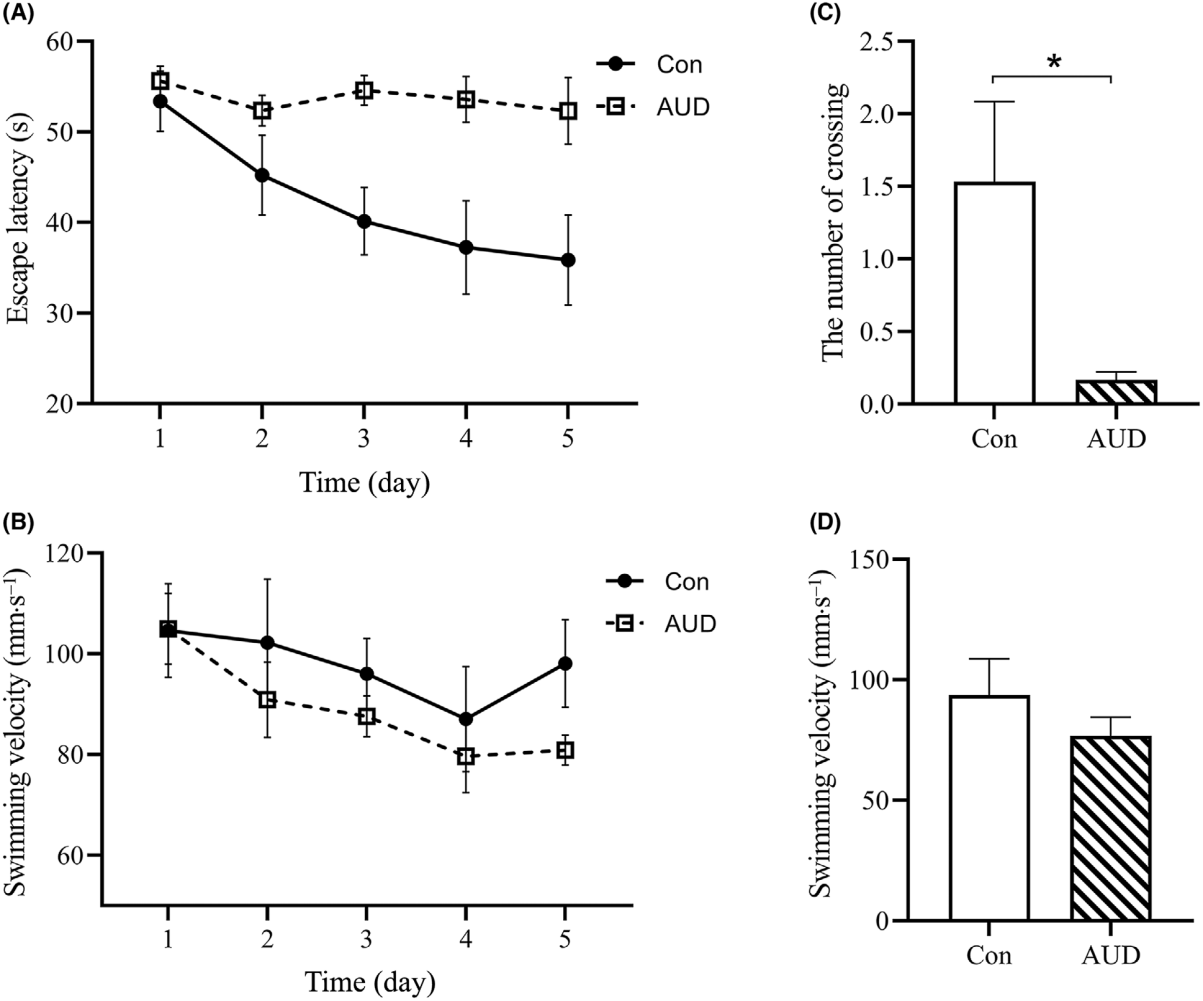
The Morris water maze test following chronic intermittent alcohol vapor (CIAV)‐treated mice. (A) The AUD mice with escape latency significantly longer than Con mice in the training phase. The training phase spanned 5 days, consisting of four trials per day. Escape latency indicates the time taken by mice to locate the submerged platform (*n* = 10). (B) There was no significant difference in swimming velocity between AUD mice and Con mice in the training phase (*n* = 10). (C) The Con mice exhibited a significantly higher number of target platform location crossings significantly compared to the AUD mice in the spatial probe test. The spatial probe test consisted of four trials per day and per animal, conducted over a duration of 1 day. Notably, no platform is present at the designated location (*n* = 10). (D) There was no significant difference in swimming velocity between AUD mice and Con mice in the spatial probe test (*n* = 10). The data are represented as mean ± SEM and analyzed using a two‐way ANOVA for repeated measurements (A,B) and unpaired Student's *t*‐test (C,D). *P < 0.05 indicates statistical significance.

Subsequently, we employed the MWM test to assess hippocampus‐related spatial learning and memory levels in both Con and AUD groups, aiming to ascertain any potential impairments in learning and memory abilities among AUD mice. During the training phase, which lasted for 5 days, with four trials per day and per animal, the escape latency (the time it took to find the submerged platform) and swimming velocity were analyzed using a two‐way ANOVA for repeated measurements with Greenhouse–Geisser correction. There was a significant group effect (Con and AUD groups; *F*(1, 18) = 21.922, *P* < 0.001) and day effect (1–5 days; *F*(4, 72) = 3.449, *P* = 0.012) on the escape latency. The escape latency was significantly decreased in the Con group compared to the AUD group (LSD *post‐hoc* comparison, *P* < 0.001). There was a significant day effect in swimming velocity (*F*(4, 72) = 3.857, *P* = 0.007), but no significant group effect and interaction effect (group × day) were detected (Fig. 3A,B).

During the spatial probe test (1 day, four trials per day and per animal), the number of target platform location crossings (no platform) was significantly decreased in the AUD group compared with the Con group (unpaired Student's *t*‐test, *t* = −2.451, *P* = 0.043). There were no significant differences between the Con group and the AUD group in terms of swimming velocity (unpaired Student's *t*‐test, *t* = −1.105, *P* > 0.05) (Fig. 3C,D).

Swimming velocity was evaluated to eliminate any bias caused by visual and motor deficits. Upon comparing the two groups, no significant differences were observed. Therefore, our findings indicate a decline in learning and memory abilities among AUD mice.

### No changes of anxiety‐like behavior and spontaneous locomotor activity after aerobic exercise intervention

**Fig. 4:**
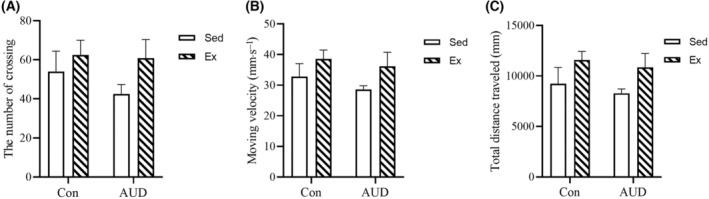
The open field test after aerobic exercise intervention. (A) There were no significant differences in the number of crossings among the four groups (*n* = 6). (B) There were no significant differences in the moving velocity among the four groups (*n* = 6). (C) The total distance traveled by the mice in the exercise (Ex) group was longer than that of the mice in the sedentary (Sed) group (*n* = 6). The data are represented as mean ± SEM and analyzed using a two‐way ANOVA.

Following the aerobic exercise intervention, anxiety‐like behavior and spontaneous locomotor activity were assessed in the Con‐Sed, Con‐Ex, AUD‐Sed, and AUD‐Ex groups using the OFT. The OFT was utilized to assess the anxiety state of mice in order to confirm that the exercise intensity did not induce stress and affect cognitive performance. The floor of the open field device was divided into nine grids, and a grid‐crossing event was recorded once when all four limbs of a mouse crossed a grid line. There were no significant differences in the number of crossings and moving velocity among the four groups (two‐way ANOVA with LSD *post‐hoc* comparison, *P* > 0.05). The study found a significant exercise effect (*F*(1, 36) = 4.447, *P* = 0.048) and AUD effect (*F*(1, 36) = 0.529, *P* = 0.47) on the total distance traveled in the Con‐Sed, Con‐Ex, AUD‐Sed, and AUD‐Ex groups. However, no significant interaction effect (exercise × AUD) was observed. This suggests that the exercise intervention enhanced certain motor function abilities in the mice, while it did not affect the mice's anxiety‐like behavior and spontaneous locomotor activity. (Fig. 4A–C). The exercise intensity may not induce additional stress in mice, thereby mitigating potential adverse effects on their learning and memory capacity.

### Improvement of learning and memory in AUD mice treated with exercise

**Fig. 5:**
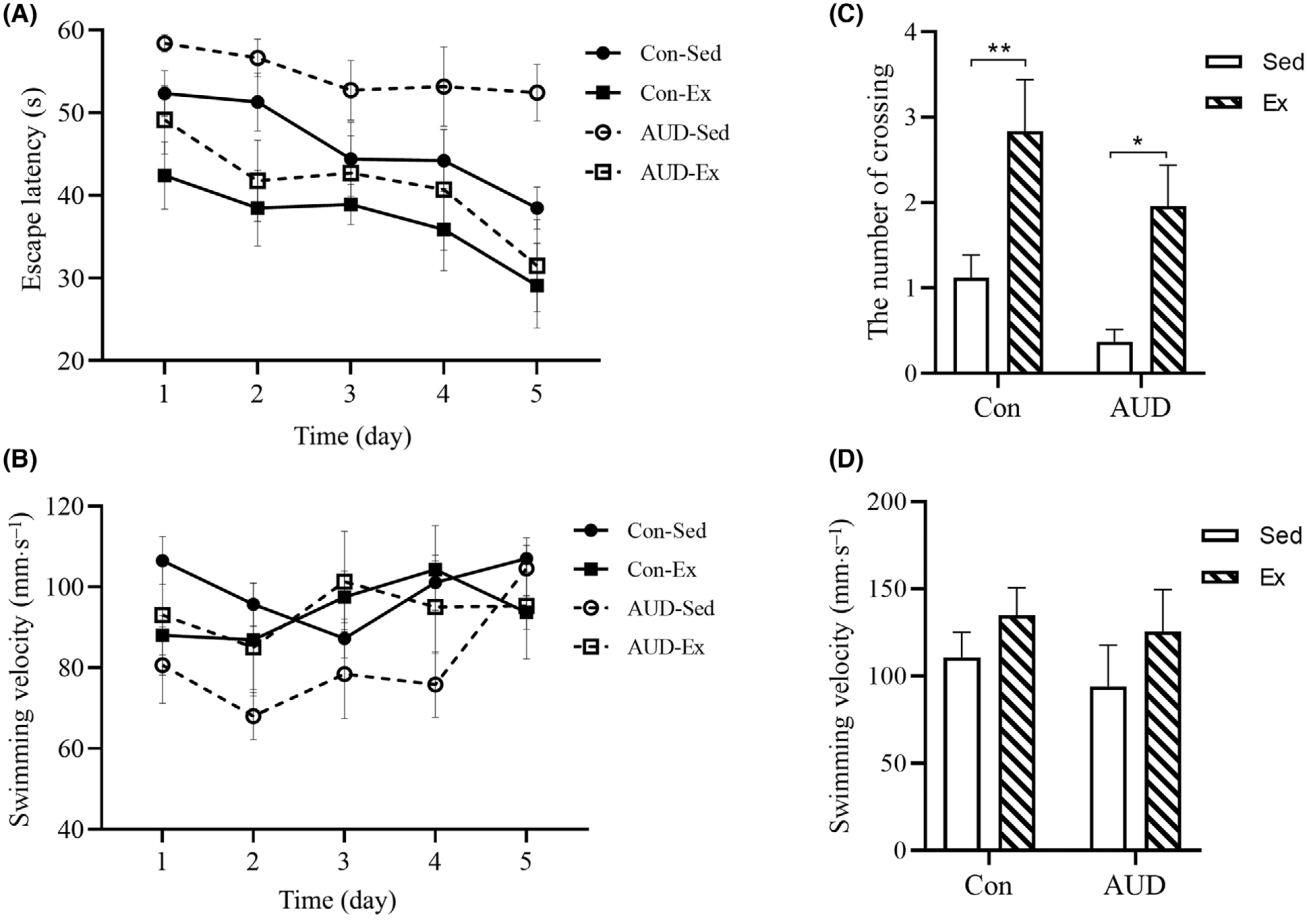
The Morris water maze test in mice treated with exercise. (A) The mice with escape latency in alcohol use disorder and sedentary (AUD‐Sed) group was significantly longer than the mice in the alcohol use disorder and exercise (AUD‐Ex) group in the training phase (*n* = 6). (B) There was no significant difference in swimming velocity among the four groups during the training phase (*n* = 6). (C) The mice in the Ex group exhibited a significantly higher number of target platform location crossings significantly compared to the mice in Sed group during the spatial probe test (*n* = 6). (D) There was no significant difference in swimming velocity among four groups in the spatial probe test (*n* = 6). The data are represented as mean ± SEM and analyzed using a two‐way ANOVA for repeated measurements (A,B) and two‐way ANOVA (C,D). *P < 0.05 and **P < 0.01 indicates statistical significance.

Following OFT, MWM was devised to examine the impact of exercise intervention on cognitive performance in mice. During the training phase, a significant group effect (Con‐Sed, Con‐Ex, AUD‐Sed, and AUD‐Ex groups) was observed using a two‐way repeated‐measures ANOVA with Mauchly's test of sphericity (*F*(3, 20) = 8.736, *P* = 0.01). Additionally, a significant day effect (1–5 days) was detected in the escape latency (*F*(4, 80) = 6.351, *P* < 0.001). However, no significant interaction effect (group × day) was found. The escape latency was significantly increased in the AUD‐Sed group compared to the Con‐Sed group (LSD *post‐hoc* comparison, *P* = 0.03). However, it was significantly decreased in the AUD‐Ex group compared to the AUD‐Sed group (LSD *post‐hoc* comparison, *P* = 0.001). There was no significant day effect, group effect, or interaction effect (group × day) on swim velocity (Fig. 5A,B).

During the spatial probe test, a significant exercise effect (exercise/no‐exercise; two‐way ANOVA, *F*(1, 20) = 15.705, *P* < 0.001) was detected in the number of target platform location crossings for the Con‐Sed, Con‐Ex, AUD‐Sed, and AUD‐Ex groups. However, no significant AUD effect (AUD‐treated/no‐AUD‐treated) or interaction effect (exercise × AUD) was found. The number of crossings was significantly higher in the AUD‐Ex group compared to the AUD‐Sed group (two‐way ANOVA with LSD *post‐hoc* comparison, *P* = 0.014). There was no significant exercise effect, AUD effect, or interaction effect (exercise × AUD) on swim velocity (Fig. 5C,D). The results showed that exercise was able to improve the learning and memory of AUD mice.

### Increased hippocampal weight and BDNF in AUD mice treated with exercise

**Fig. 6:**
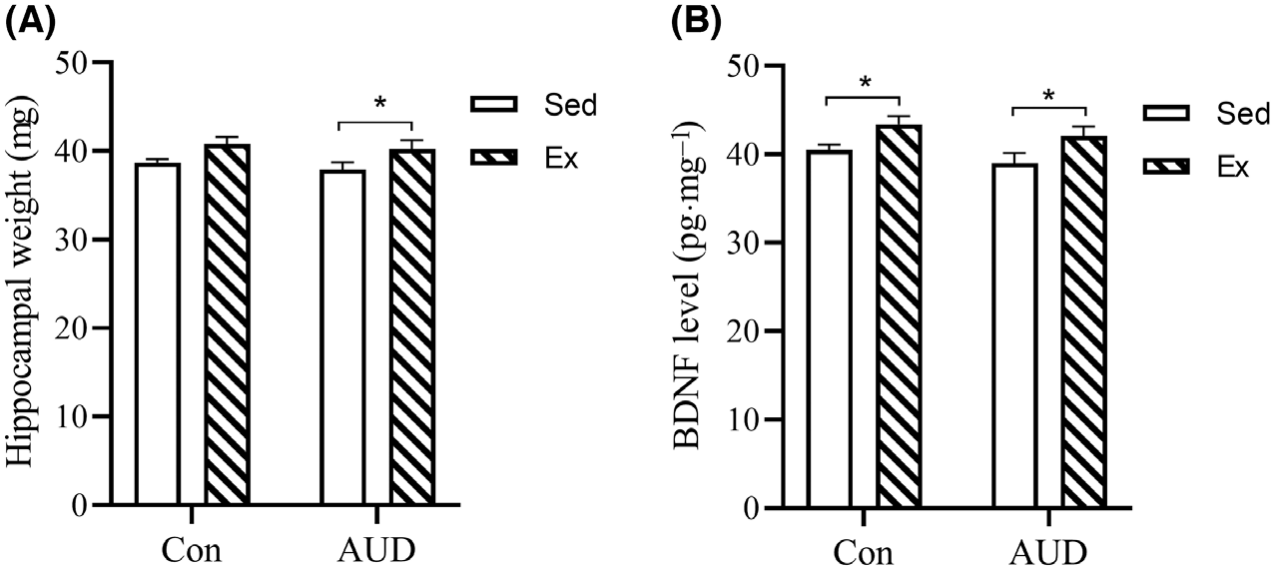
Hippocampal weight and brain‐derived neurotrophic factor (BDNF) levels in mice treated with exercise. (A) The hippocampal weight of mice in the AUD‐Sed group was significantly lower compared to that of the ADU‐Ex group mice (*n* = 10). (B) The levels of BDNF in the hippocampus of the mice in the Ex groups were significantly higher than those in the Sed groups. The BDNF level (pg·mg^−1^) was calculated as sample BDNF concentration (pg·mL^−1^) divided by total sample protein concentration (mg·mL^−1^) (*n* = 10). The data are represented as mean ± SEM and analyzed using a two‐way ANOVA. *P < 0.05 indicates statistical significance.

Hippocampal weight and BDNF levels are closely related to learning and memory capacity. The study found a significant exercise effect on hippocampal weight in the Con‐Sed, Con‐Ex, AUD‐Sed, and AUD‐Ex groups (two‐way ANOVA, *F*(1, 36) = 8.181, *P* = 0.007). However, no significant AUD effect or interaction effect (exercise × AUD) was observed. The weights of the hippocampus were significantly increased in the AUD‐Ex group compared to the AUD‐Sed group (LSD *post‐hoc* comparison, *P* = 0.045) (Fig. 6A).

The study found a significant exercise effect (two‐way ANOVA, *F*(1, 36) = 9.720, *P* = 0.004) on the BDNF levels in the hippocampus of the Con‐Sed, Con‐Ex, AUD‐Sed, and AUD‐Ex groups. However, no significant AUD effect or interaction effect (exercise × AUD) was observed. The levels of BDNF were significantly increased in the AUD‐Ex group compared to the AUD‐Sed group (LSD *post‐hoc* comparison, *P* = 0.029) (Fig. 6B).

### Improvement of monoamine neurotransmitters in AUD mice treated with exercise

**Fig. 7:**
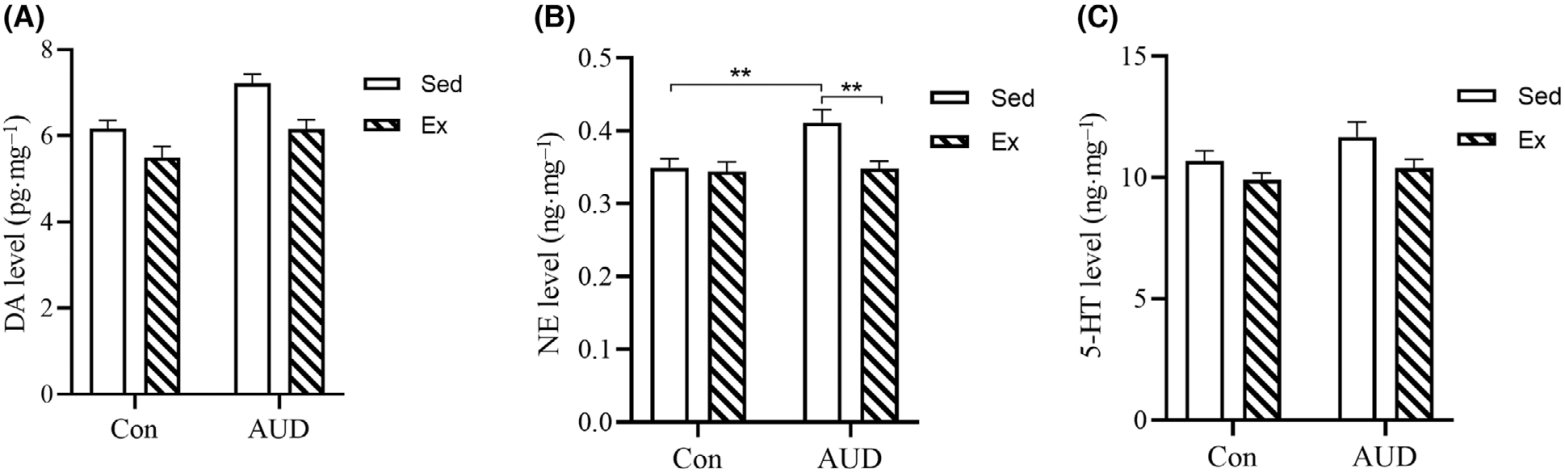
The levels of monoamine neurotransmitters from the hippocampus in mice treated with exercise. (A) Exercise reduced the levels of the dopamine (DA) in the hippocampus of mice, regardless of exposure to alcohol. The DA level (pg·mg^−1^) was calculated as sample DA concentration (pg·mL^−1^) divided by total sample protein concentration (mg·mL^−1^) (*n* = 10). (B) Exercise reduced the levels of norepinephrine (NE) in the hippocampus of AUD mice. The NE level (ng·mg^−1^) was calculated as sample NE concentration (ng·mL^−1^) divided by total sample protein concentration (mg·mL^−1^) (*n* = 10). (C) Exercise reduced the levels of the serotonin (5‐HT) in the hippocampus of mice, regardless of exposure to alcohol. The 5‐HT level (ng·mg^−1^) was calculated as sample 5‐HT concentration (ng·mL^−1^) divided by total sample protein concentration (mg·mL^−1^) (*n* = 10). The data are represented as mean ± SEM and analyzed using a two‐way ANOVA. *P < 0.05, **P < 0.01 indicates statistical significance.

The levels of monoamine neurotransmitters, including DA, NE, and 5‐HT, were analyzed using a two‐way ANOVA with LSD *post‐hoc* comparison. The study found a significant exercise effect (*F*(1, 36) = 15.348, *P* < 0.001) and AUD effect (*F*(1, 36) = 15.040, *P* < 0.001) on DA levels in the Con‐Sed, Con‐Ex, AUD‐Sed, and AUD‐Ex groups. However, no significant interaction effect (exercise × AUD) was observed (Fig. 7A).

The study found a significant exercise effect (*F*(1, 36) = 6.370, *P* = 0.016), AUD effect (*F*(1, 36) = 5.871, *P* = 0.021), and interaction effect (exercise × AUD; *F*(1, 36) = 4.574, *P* = 0.039) on the NE levels in the four groups. The results of the simple effect analysis showed that the levels of NE in the AUD‐Sed group were higher than those in the Con‐Sed group (*P* = 0.003) and the AUD‐Ex group (*P* = 0.002), respectively (Fig. 7B).

The study revealed a significant exercise effect (*F*(1, 36) = 5.574, *P* = 0.024) on the levels of 5‐HT among the four groups. However, no significant AUD effect or interaction effect (exercise × AUD) was observed (Fig. 7C).

The results showed that exercise effectively attenuated the levels of NE in the hippocampus of AUD mice, thereby ameliorating NE dysregulation. But the study found that exercise reduced DA, 5‐HT levels in the hippocampus of mice, regardless of whether or not they were exposed to alcohol.

## Discussion

Our results suggested that alcohol consumption had a detrimental impact on learning and memory, with no restoration observed even after a 2‐month withdrawal period. In contrast, an 8‐week aerobic exercise intervention underwent alcohol withdrawal not only enhanced learning and memory but also elevated the levels of BDNF and hippocampal weight, while reducing the levels of DA, NE, and 5‐HT in the hippocampus. The improvement in learning and memory capacity of AUD mice after alcohol withdrawal through aerobic exercise is possibly related to ameliorating monoamine neurotransmitter levels. These findings provide empirical evidence and data supporting the efficacy of aerobic exercise as a potential therapeutic approach for mitigating cognitive impairment associated with AUD.

### Selection of the AUD model

Given the intricate nature of diagnosing AUD, primarily reliant on behavioral criteria, establishing an animal model that encompasses all characteristics of AUD becomes exceedingly challenging. Consequently, the choice of modeling method varies depending on the study's objectives, including two‐bottle choices, CIAV, alcohol gavage, alcohol liquid diet, and others. Previous studies have reported the successful establishment of the AUD mice model using CIAV, which effectively maintains BAC levels within the range of 175–225 mg·dL^−1^ to develop the AUD characteristics [23]. Due to its high success rate in modeling, CIAV exposure is frequently employed for studying pathological molecular alterations in AUD. Thus, CIAV exposure was utilized for modeling in this study.

To further validate the efficacy of the custom‐made equipment model, the 2BC test was used as a measure of alcohol consumption [23], aiming to ascertain whether CIAV exposure leads to increased alcohol consumption (one of the diagnostic criteria for AUD) [25]. Successful performance of the alcohol consumption test requires pre‐2BC drinking training, thus necessitating 2BC prior to CIAV. Typically, mice establish a stable baseline of alcohol consumption after 4 weeks of 2‐BC drinking training; however, for a more precise and stable baseline, this study utilized an extended period of 8 weeks. Therefore, this study employed an 8‐week period of 2BC combined with a subsequent exposure to CIAV lasting for 4 weeks in order to co‐establish the AUD model.

Over recent decades, gender differences in alcohol use have diminished due to changes in the socio‐psycho‐cultural environment. In countries where the number of drinkers is increasing, both young men and women are starting to drink at an earlier age [34]. However, studies have found that men consume more alcohol than women, and drink more frequently [35, 36]. For instance, in South Korea the rate of high‐risk drinking among men (37%) is approximately five times higher than that among women (8%) [37]. Moreover, the prevalence of alcohol‐related problems is higher among men. Research indicates that men have a higher lifetime prevalence of alcohol use disorders (36.0%) compared to women (22.7%) [38]. Given the higher risk of drinking and the prevalence of alcohol use disorders in men, and due to limited experimental capacity that cannot accommodate both male and female mice simultaneously, this study focused exclusively on male mice.

It is worth noting that the transition from single to group housing during the CIAV exposure phase may impose stressful pressure on mice, potentially leading to cognitive deficits. Therefore, a period of group adaptation was implemented between 2BC and CIAV in order to mitigate any potential impact on the experimental results. The acclimatization to group housing was assessed based on changes in weight and behavioral indicators (e.g. aggressive behavior, coat color, etc.). During the preadaptation phase, the body weight results indicated that control mice had a significantly higher weight than AUD mice, which could be attributed to discomfort and irritability resulting from alcohol abstinence in AUD mice. And the mice developed fighting behavior. We repositioned the combatant rats, altered their partners, resulting in a notable reduction in aggressive behavior. During the postadaptation stage, there was a significant increase in body weight growth trend for AUD mice, and the gap between their body weights and those of control mice gradually decreased. Additionally, there was a significant decrease in the fighting behavior among mice, indicating successful group adaptation (Fig. S1).

### Changes in mice by CIAV‐treated

#### AUD modeling success

To mitigate potential stress and inflammation caused by blood collection in experimental mice, which could subsequently impact the behavioral testing outcomes, sentinel mice were employed for monitoring BAC levels instead of performing blood collection on all experimental subjects. Our findings demonstrated that BAC were maintained within the range of 200–220 mg·dL^−1^, and a significant increase in alcohol consumption was observed in the AUD group. It is worth noting that many studies utilize intraperitoneal injection of alcohol and 4‐methylpyrazole prior to exposure to alcohol vapor in experimental animals. However, pyrazole may induce central nervous system depressant side effects unrelated to alcohol metabolism [26], as well as potentially affecting mitochondrial morphology and function [27]. Therefore, to avoid potential cognitive effects associated with pyrazole administration, only intraperitoneal administration of alcohol was employed. According to previous studies on repeated chronic ethanol exposure [24], mice exhibit a significant increase in alcohol consumption, while there is no notable change in sucrose solution consumption. These findings demonstrate the selectivity of mice for alcohol and confirm the validity of the inhalation device used in this study.

#### Learning and memory impairment

Due to the strong aversion of mice to water, the MWM is able to mitigate the impact of decreased locomotor motivation that may occur during terrestrial testing [39]. Thus, following CIAV exposure we utilized the MWM to evaluate the learning and memory abilities of AUD mice for further confirming the successful AUD modeling. A previous study has reported impaired learning abilities, encoding processes, contextual memory, and autonoetic consciousness in recently detoxified alcoholic inpatients [40]. Consistent with this research, our study also demonstrates a significant decrease in learning and memory among AUD mice. Therefore, these results further validate the successful construction of the AUD model. Furthermore, there was no significant difference in swimming velocity between AUD and control mice, indicating that alcohol withdrawal has a minimal impact on MWM motor performance. It is worth noting that, to minimize potential interference from withdrawal reactions on experimental outcomes, according to a previous study, the MWM test was conducted 4 days after the last alcohol consumption session [41].

### Changes in mice by exercise‐treated

#### Learning and memory improvement by exercise in AUD following withdrawal

Treadmill running in mice is often considered a form of forced exercise and has been proven to increase biomarkers of stress response in rodents [42]. To investigate whether long‐term exercise leads to stress responses and induces anxiety‐like behaviors in mice, following completion of the aerobic exercise we evaluated the impact of AUD and exercise‐related factors on spontaneous activity and anxiety levels in mice using the OFT. Our results demonstrated that these variables did not yield a significant influence on spontaneous activity or anxiety levels. This suggests that the exercise intensity employed does not provide additional stress or adversely affect learning and memory in mice.

Alcohol consumption induces memory impairment, while exercise can enhance learning and memory through cognitive function improvement, neuroprotection promotion, and reversal of the alcohol‐induced inhibition of neurogenesis [43, 44]. From the results, it was clear that aerobic exercise effectively improved learning and memory in mice with AUD. However, some researchers found that exercise intervention fails to improve habitual learning and working memory in alcohol‐exposed mice [45]. These varying effects of exercise treatment may be attributed to the different severity of alcohol‐induced brain damage in the experiments, and exercise intervention protocols. The alcohol‐induced brain damage may depend on various variables, such as alcohol exposure time, gender, and age [43], while the effect of exercise intervention on cognition may be associated with time, intensity, and training type [46]. Additionally, the benefits of treadmill training may be influenced by the circadian rhythm of mice. One study discovered that mice derive greater benefits from training either during the day or at night, as opposed to training at dawn [45]. In our study, the mice were subjected to a circadian cycle with lights out at 14:00 h and lights on at 02:00 h. It was observed that the mice became increasingly active during the lights out period, which is commonly associated with improved cognitive performance [45]. In this study, the mice were all treated with the exercise intervention at the same time of night (starting at 16:00 h), mitigating potential adverse effects of the circadian rhythm on learning and memory.

There is growing evidence that the BDNF level plays an important role in learning and memory, synaptic plasticity, and long‐term potentiation [46]. However, a previous study reported that alcohol consumption leads to reductions in neurogenesis, hippocampal volume, and BDNF levels [43]. It has been known that physical exercise can increase synaptic plasticity, elevate BDNF levels, and improve learning and memory [47, 48]. In this study the results demonstrated exercise treatment may improve learning and memory abilities in AUD mice by increasing hippocampal weights and BDNF levels. Notably, some studies reported that alcohol exposure damages nerve cells and decreases levels of BDNF [49]. Therefore, further research is needed to determine whether exercise can increase BDNF levels and learning and memory abilities by repairing nerve cells.

#### Improvements in learning and memory may be related to exercise ameliorating neurotransmitters

##### Dopamine

Negative emotions and cravings have been identified as significant predictors of relapse in individuals with AUD. Alcohol craving is considered a defining characteristic of AUD and is positively associated with the severity of alcohol dependence [50, 51]. Dopamine, a crucial neurotransmitter, plays a pivotal role in triggering relapse, while its levels influence the intensity of cravings. The study by Hirth *et al*. [52] demonstrated that during alcohol withdrawal and long‐term abstinence, the DA system exhibits dynamic changes, characterized by a hypodopaminergic state during acute withdrawal and a hyperdopaminergic state during prolonged abstinence. This means that DA levels decrease at the beginning of acute withdrawal and increase after 6 days into prolonged withdrawal. Furthermore, previous research investigating the change of DA levels in addicted and readdicted rats has reported that prior consumption of addictive substances disrupts the neurohumoral regulation in the brain, leading to an excessive release of DA that persists at elevated levels even after detoxification [53]. These findings suggest a strong correlation between dopamine levels and AUD following a period of abstinence from alcohol. Our research results found that, after a period of abstinence, the DA levels in the hippocampus of AUD mice were significantly higher than those in control mice, aligning with prior studies. This suggests that AUD may disrupt the DA system in the hippocampus of mice, resulting in an aberrant increase in DA release.

Li and Gao [54] found that moderate levels of DA can induce long‐term potentiation by activating the D_1_ receptor (D_1_R), thereby enhancing cognition. However, excessive dopamine can lead to the generation of neurotoxins and impair cognitive function [55]. Moreover, overstimulation of the DA‐D_1_R system impairs spatial learning and memory in rats [56]. Therefore, the present study revealed that impaired learning and memory abilities in AUD mice following alcohol withdrawal may be associated with aberrant DA levels, as compared to control mice.

It has been discovered in a study that exercise can exert a bidirectional regulatory effect on DA, promoting its synthesis, secretion, and release while also improving its catabolism and metabolism [19]. And exercise specifically increased the conversion rate of dopamine, particularly through enhanced DA catabolism and metabolism [19]. This suggests a gradual decrease in DA levels following exercise cessation. Additionally, long‐term aerobic exercise has been reported to ameliorate the blocked release pathway of dopamine in addicts and increase D_2_ receptor levels for DA, thereby normalizing the reward pathway and enhancing dopaminergic homeostasis in the midbrain [57]. The observed reduced DA levels in mice, regardless of whether or not they were exposed to alcohol, may be related to the fact that exercise increases DA conversion and increases D_2_ receptor levels. Given the close association between DA levels and learning and memory abilities, it is plausible to suggest that the observed enhancement in learning and memory abilities following alcohol withdrawal in AUD mice may be attributed to exercise‐mediated decrease in hippocampal DA levels.

##### Norepinephrine

Intrahippocampal norepinephrine (NE) primarily originates from the locus coeruleus (LC) [58]. Prolonged usage of addictive substances, such as alcohol and nicotine, results in an upregulation of norepinephrine signaling during withdrawal [59]. The results of the present study revealed a significant elevation in NE levels within the hippocampus of AUD mice after long‐term alcohol withdrawal compared to control mice, which may indicate the presence of an aberrant LC‐mediated mechanism for norepinephrine supply in the hippocampus of individuals with AUD, even in the absence of alcohol exposure. It may be attributed to hyperexcitation of NE neurons and abnormal elevation in DA levels. The previous studies indicated that alcohol consumption led to an upregulation of β‐endorphin (β‐EP) within the endogenous opioid peptide system [60]. Chronic exposure to alcohol gradually induces adaptive changes, subsequently resulting in reduced production of endogenous opioid peptides. Following alcohol withdrawal, endogenous opioid peptides levels may not return to their initial state or even become lower than normal due to diminished responsiveness. Given that the synthesis, secretion, and release of endogenous opioid peptides are inversely proportional to the activity levels of NE‐ergic neurons [61, 62], long‐term alcohol withdrawal may lead to an aberrant endogenous opioid peptide system. This system, having adapted to chronic alcohol consumption, disrupts the regulatory mechanisms of NE neurons, resulting in their overexcitation. In addition, it has been reported that DA in the dorsal hippocampus primarily originates from release by the LC [63]. Given that DA serves as a precursor for NE synthesis, the aberrant elevation of NE in the hippocampus could potentially be associated with heightened DA content within this brain region.

Norepinephrine neurons located in the LC have been implicated in various cognitive functions. Overstimulation of these neurons has been associated with working memory deficits, while excessive levels of NE can impair working memory in both the peripheral and central systems [58, 64, 65]. Animal and human studies indicated that norepinephrine affects working memory in a dose‐dependent, “inverted U‐shaped” manner (i.e. excessively high or low levels of NE can impair working memory) [66, 67]. Therefore, these findings suggested a potential association between aberrant NE levels and impaired learning and memory abilities. The present study revealed that AUD mice exhibited excessive NE levels and experienced reduced learning and memory capacities following alcohol withdrawal compared to control mice. These results imply a possible relationship between NE levels and the cognitive performance of AUD mice.

The present study revealed a decrease in NE levels in exercise‐treated AUD mice, potentially indicating a correlation between exercise‐induced inhibition of NE release and increased β‐EP concentrations, as well as modulation of neural pathways. Previous research has demonstrated that exercise can modulate the HPA‐CORT neural pathway, leading to reduced NE synthesis rates [19]. Furthermore, it has been shown that moderate‐ to high‐intensity aerobic exercise can elevate β‐EP concentrations [68, 69].

##### 5‐Hydroxytryptamine

Drug addiction is closely associated with the release of the neurotransmitter 5‐HT and the activation of its receptors, which primarily influences the dopamine system. Elevated 5‐HT levels in the brain can stimulate DA release and subsequently increase DA levels [70]. The activation of 5‐HT receptors is closely linked to DA release, with five receptors enhancing DA release and one receptor suppressing it [71].

It has been observed that the concentration of 5‐HT is associated with the maintenance or improvement of memory ability, and a decrease in its level impairs spatial memory [72]. In contrast to previous studies, our finding revealed that the levels of 5‐HT in the hippocampus of AUD mice following long‐term alcohol withdrawal were slightly elevated, while their learning and memory abilities were reduced. However, following alcohol withdrawal, an 8‐week regular aerobic exercise regimen was found to reduce intrahippocampal 5‐HT levels and improve learning and memory abilities in AUD mice. This suggests that exercise may augment cognitive functions by decreasing intrahippocampal 5‐HT concentrations. The change of exercise for 5‐HT could potentially be linked to improved homeostasis of DA and 5‐HT. Previous studies have demonstrated a disruption in the balance between 5‐HT and DA within the central nervous system of individuals with AUD [20]. Furthermore, it has been suggested that exercise‐induced secretion of β‐EP in the hippocampus may contribute to restoring this balance by attenuating central nervous system activity associated with DA [73, 74]. In line with these findings, our study observed alterations in both DA and 5‐HT levels within the hippocampus of AUD mice following exercise intervention, indicating that exercise holds potential for ameliorating the imbalance of DA and 5‐HT.

Notably, the studies regarding the effects of exercise on 5‐HT levels have discrepancies. Some studies had reported increased brain 5‐HT concentrations following wheel‐running exercises in mice [75], while others indicated that downregulation of hippocampal 5‐HT levels enhances passive avoidance learning, as well as fear memory acquisition during physical activity [76]. Therefore, further investigations are warranted to explore the potential of exercise in enhancing learning and memory through modulation of 5‐HT levels.

In conclusion, the aberrant hippocampal DA, NE, and 5‐HT levels were observed in AUD mice following alcohol withdrawal, concomitant with persistent impairments in learning and memory function. Notably, aerobic exercise was found to ameliorate the cognitive deficits in AUD mice, potentially attributed to the modulation of hippocampal DA, NE, and 5‐HT levels.

It is worth noting that there are several aspects that require discussion. First, previous research observed that stress activates the HPA system in organisms, and CORT mediation within this neural pathway leads to an elevation in both DA and NE metabolite levels within the hippocampus [19], indicating stress can cause increased DA and NE levels in the hippocampus. While mice in the current experiment may experience stress during the MWM test and exercise, leading to an abnormal increase in monoamine neurotransmitters, brain dissections were performed 1 day after the last exercise session (i.e. 5 days with the final water maze test). Considering the rapid degradation of monoamine neurotransmitters following their release [77], it is plausible that the abnormal increase in monoamine neurotransmission observed in AUD mice cannot be solely attributed to stress.

In addition, some previous studies reported that DA, NE, and 5‐HT levels from the hippocampus were higher in rats following exercise compared to normal rats [78, 79]. However, our results demonstrated a reduction in levels of DA, NE, and 5‐HT in normal mice following exercise. This discrepancy may be attributed to a decrease in running speed from 12 m/s to 10 m/s during the last 4 days and a 24‐h gap between exercise and dissection. Previous research had shown that acute postexercise leads to an increase in DA levels, which return to baseline after 24 h. Conversely, prolonged exercise training decreases basal DA levels, resulting in diminished elevation of DA following a single exercise session [18]. Some evidence suggested that concentrations of DA, 5‐HT, and NE may be lower in trained rodent brain regions compared to nontrained conditions [80, 81, 82]. Furthermore, long‐term continuous exercise training has been found to result in decreased levels of DA and NE during the postpractice quiet state compared to pretraining [19, 83]. In this experiment, it was observed that AUD mice, who had been chronically exposed to alcohol and underwent behavioral tests after 8 weeks of exercise, exhibited an increased risk to unexpected mortality. As a precautionary measure, the running speed was slightly reduced, which potentially resulted in a decrease in peak dopamine release in mice. And at 24 h postdissection, DA may have undergone breakdown and metabolism to basal levels, which baseline following exercise lower than nonexercise treatment, leading to slightly lower DA levels in normal mice within the exercise group. Furthermore, this breakdown could also be associated with marginally decreased levels of NE and 5‐HT. However, it is important to note that these findings did not impact the final outcomes.

### Limitations

There are some limitations of this study. First, the method of reading was used to record the alcohol consumption of mice during the two‐bottle choice session, which is not very accurate due to the presence of a small amount of liquid in the drinking spout. Reading was changed to weighing at a later time in the experiment and it did not affect the conclusions. Second, the present study mainly investigated the content of monoamine neurotransmitters in the hippocampus of AUD mice, but the nervous system is complex and diverse, and there are still brain regions other than the hippocampal brain region that affect learning and memory abilities. Third, in our study on BDNF, DA, NE, and 5‐HT, we did not differentiate whether the vesicles of these molecules were in a state of storage or release. Lastly, only male mice were investigated in this study, while alcohol consumption can have different impacts on different genders. Therefore, in future studies the mechanisms of the impacts of exercise interventions on AUD can be investigated in different brain regions and genders, and weighing should be used to record the amount of alcohol consumed to ensure accuracy.

## Conclusion

**Fig. 8:**
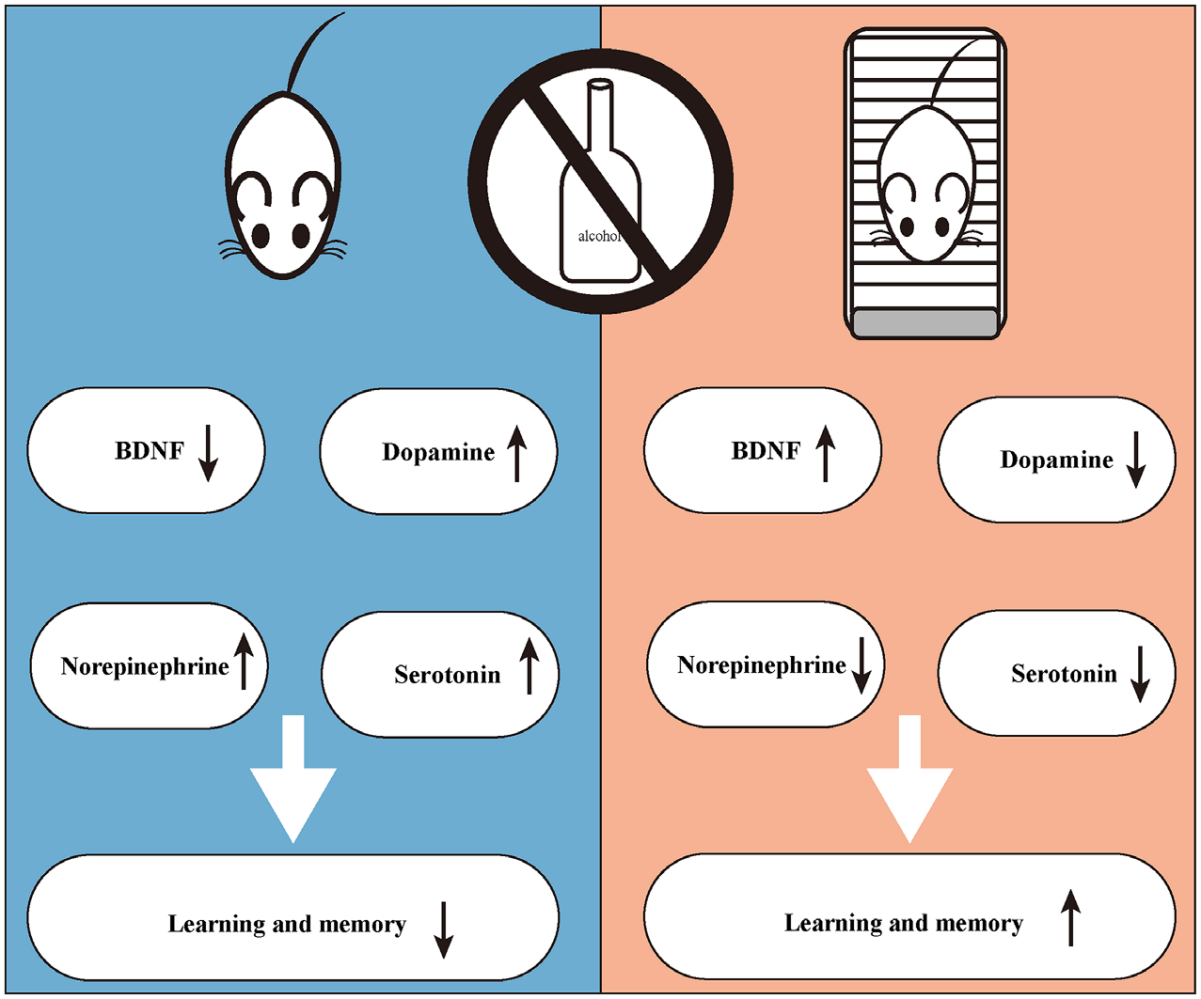
A model demonstrating how exercise affects monoamine neurotransmitters and BDNF levels in AUD mice following abstinence, thereby influencing learning and memory capabilities.

In this study we found that the monoamine neurotransmitter system of AUD mice exhibited disorganization, resulting in an aberrant increase in DA, NE, and 5‐HT release within the hippocampus. These alterations may impair learning and memory abilities. However, aerobic exercise exerted a positive impact by reducing DA, NE, and 5‐HT levels in the hippocampus, while increasing BDNF expression and promoting hippocampal weight gain. Ultimately, exercise intervention enhances learning and memory abilities in AUD mice (Fig. 8). Briefly, the efficacy of aerobic exercise in enhancing learning and memory ability within AUD may be related to its capacity for ameliorating disruptions in monoamine neurotransmitters within the hippocampus, elevating BDNF levels, and augmenting hippocampal weight.

## Conflict of interest

The authors declare no conflicts of interest.

## Author contributions

ZL and Z‐GG conceived and designed the project; ZL, M‐XH, S‐PX, M‐ZZ, and Y‐QD acquired the data; ZL analyzed and interpreted the data; ZL, M‐XH, and Z‐GG wrote the article.

## Supporting information


**Data S1.** Supplementary results.
**Fig. S1.** The body weight changes in mice.

## Data Availability

The data that support the findings of this study are available from the corresponding author upon reasonable request.
