# Supplementary material for: Benefits of exercise on cognitive impairment in alcohol use disorder following alcohol withdrawal
Source: FEBS Open Bio. 2024 Jul 25;14(9):1540–58. doi: 10.1002/2211-5463.13865 (PMC11492329; doi:10.1002/2211-5463.13865)
Supplement: Supplementary file 1 — Data S1. Supplementary results. Fig. S1. The body weight changes in mice. [file FEB4-14-1540-s001.pdf]

Supplementary Information

Benefits of Exercise on Cognitive Impairment in Alcohol Use Disorder Following Alcohol Withdrawal

Zhen Lyu[1 2], Zhi-Gang Gong[1*], Si-Ping Xin[1], Mao-Zhong Zou[1], Yu-Quan Ding[1]

1. Key Lab of Aquatic Sports Training Monitoring and Intervention of General Administration of Sport of China, Faculty of Physical Education, Jiangxi Normal University, No.99 Ziyang Avenue, Nanchang 330022, Jiangxi, China

2. Shanghai University of Sport, No.399 Changhai Road, Shanghai 200438, China

Supplementary Results

Body weights of mice were measured at a fixed time each week, and analyzed using a two-way ANOVA for repeated measurements with Greenhouse-Geisser correction. A significant week effect (1-16 weeks; $F = 486.750$，$p < 0.001$) and interaction effect (group x week; $F = 5.144$，$p < 0.001$) on the body weight before and after AUD modeling were observed, but no significant group effect (Con and AUD groups) was detected. Simple effect analysis revealed that the body weights in the AUD group were significantly decreased during week 9 ($p = 0.008<0.01$) and week 10 ($p = 0.025<0.05$), compared to the Con group (Supplementary Figure 1A).

The body weight before and after mice treated with exercise was analyzed using a two-way repeated measures ANOVA with Greenhouse-Geisser correction. A significant week effect(17-28 weeks; $F = 97.119$，$p < 0.001$) and interaction effect (group x week; $F = 2.443$，$p = 0.006 < 0.01$) on the body weight before and after mice treated with exercise were observed. However, no significant group effect (Con-Sed, Con-Ex, AUD-Sed, and AUD-Ex groups; $F = 0.478$，$p= 0.700 > 0.05$) was detected. Further simple effect analysis showed that no significant differences in the body weight among the four groups at any given time point, suggesting that exercise interventions do not affect body weight in mice (Supplementary Figure 1B).

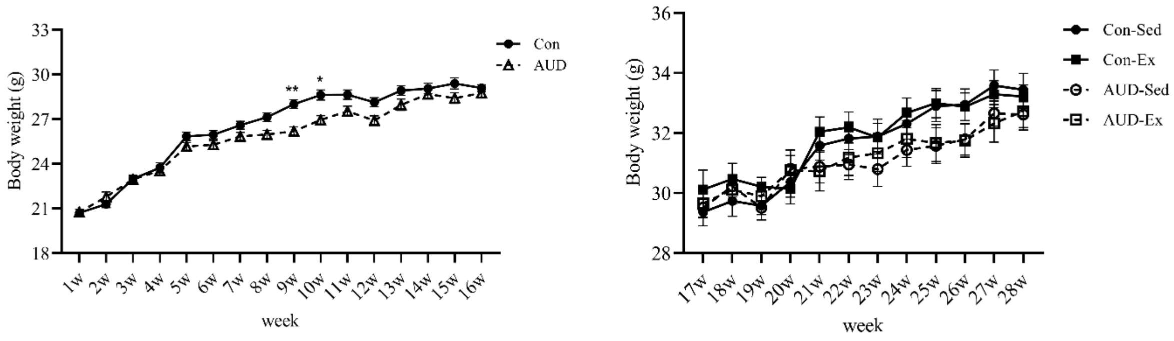

Supplementary Figure 1 The body weight changes in mice. (A) Body weight changes before and after AUD modeling. (B) Body weight changes before and after mice treated with exercise. 1w is week 1 after acclimatization rearing. *p < 0.05, **p < 0.01 indicates statistical significance
